# C5a Activates a Pro-Inflammatory Gene Expression Profile in Human Gaucher iPSC-Derived Macrophages

**DOI:** 10.3390/ijms22189912

**Published:** 2021-09-14

**Authors:** Jacquelyn C. Serfecz, Afsoon Saadin, Clayton P. Santiago, Yuji Zhang, Søren M. Bentzen, Stefanie N. Vogel, Ricardo A. Feldman

**Affiliations:** 1 Department of Microbiology and Immunology, University of Maryland School of Medicine, Baltimore, MD 21201, USA; Jackieserfecz@gmail.com (J.C.S.); ASaadin@som.umaryland.edu (A.S.); SVogel@som.umaryland.edu (S.N.V.); 2 The Solomon H. Snyder Department of Neuroscience, Johns Hopkins University School of Medicine, Baltimore, MD 21205, USA; csanti10@jhmi.edu; 3 Department of Epidemiology and Public Health, University of Maryland, Baltimore, MD 21201, USA; yuzhang@som.umaryland.edu (Y.Z.); SBentzen@som.umaryland.edu (S.M.B.)

**Keywords:** gaucher disease, induced pluripotent stem cells (iPSC), macrophages, complement, C5a, inflammation, TNF-alpha, gene arrays, GBA1, β-glucocerebrosidase

## Abstract

Gaucher disease (GD) is an autosomal recessive disorder caused by bi-allelic *GBA1* mutations that reduce the activity of the lysosomal enzyme β-glucocerebrosidase (GCase). GCase catalyzes the conversion of glucosylceramide (GluCer), a ubiquitous glycosphingolipid, to glucose and ceramide. GCase deficiency causes the accumulation of GluCer and its metabolite glucosylsphingosine (GluSph) in a number of tissues and organs. In the immune system, GCase deficiency deregulates signal transduction events, resulting in an inflammatory environment. It is known that the complement system promotes inflammation, and complement inhibitors are currently being considered as a novel therapy for GD; however, the mechanism by which complement drives systemic macrophage-mediated inflammation remains incompletely understood. To help understand the mechanisms involved, we used human GD-induced pluripotent stem cell (iPSC)-derived macrophages. We found that GD macrophages exhibit exacerbated production of inflammatory cytokines via an innate immune response mediated by receptor 1 for complement component C5a (C5aR1). Quantitative RT-PCR and ELISA assays showed that in the presence of recombinant C5a (rC5a), GD macrophages secreted 8–10-fold higher levels of TNF-α compared to rC5a-stimulated control macrophages. PMX53, a C5aR1 blocker, reversed the enhanced GD macrophage TNF-α production, indicating that the observed effect was predominantly C5aR1-mediated. To further analyze the extent of changes induced by rC5a stimulation, we performed gene array analysis of the rC5a-treated macrophage transcriptomes. We found that rC5a-stimulated GD macrophages exhibit increased expression of genes involved in TNF-α inflammatory responses compared to rC5a-stimulated controls. Our results suggest that rC5a-induced inflammation in GD macrophages activates a unique immune response, supporting the potential use of inhibitors of the C5a-C5aR1 receptor axis to mitigate the chronic inflammatory abnormalities associated with GD.

## 1. Introduction

Gaucher disease (GD) is the most frequent lipid storage disease, affecting about 1 in 1000 individuals in the Ashkenazi Jewish population, and 1 in 50,000 individuals in the general population [1]. The GD genotypes are caused by a variety of more than 500 unique mutations distributed along the 7.6 kb *GBA1* gene, including recombinations with the adjacent *GBAP1* pseudogene [2,3,4]. *GBA1* encodes the lysosomal enzyme β-glucocerebrosidase (GCase), which catalyzes the hydrolysis of glucosylceramide to glucose and ceramide. GCase deficiency results in a large accumulation of glucosylceramide (GluCer), particularly in the lysosomes of GD macrophages [5,6], due to their inability to break down GluCer present on the membrane of phagocytosed aged red blood cells. Other leukocytes are also affected in GD, leading to chronic inflammation and in some cases, development of B-cell and T-cell lymphomas [7,8,9,10,11]. The accumulation of GluCer and its deacylated derivative glucosylsphingosine (GluSph) has also been reported in iPSC-neuronal cells derived from GD patients [12,13]. The abnormal elevation of glucosylsphingolipids in type 1 GD results in anemia, excessive bruising, hepato-splenomegalia, and bone pathologies including reduced bone mineral density, avascular necrosis, and bone infarctions [14,15]. In neuronopathic, types 2 and 3 GD, there is fatal neurodegeneration in addition to visceral abnormalities [15]. At the subcellular level, tubular structured lysosomes form in giant macrophages that become engorged with undigested lipid. Pathological macrophages have elevated expression of cytokines, chemokines, and complement, leading to chronic systemic inflammation [16,17,18]. Currently, the primary treatment for the visceral abnormalities of GD is enzyme replacement therapy (ERT) with recombinant glucocerebrosidase (rGCase), and more recently, substrate reduction therapy (SRT) with GluCer synthase inhibitors [1,19]. However, ERT and SRT do not completely prevent inflammation, the increased risk of malignancies, or the hepatic fibrosis associated with GD [20,21,22,23,24,25,26]. Complement inhibitors have shown recent promise as an adjunctive therapy for GD in murine models, but their biological mechanisms of action remain unknown [21,27].

The complement system consists of more than 20 proteins that interact with each other to produce inflammatory mediators, promote phagocytosis, and form the membrane attack complex (MAC). Mouse models of GD with pharmacologically induced GCase deficiency, and mice with *Gba1* mutations, exhibit tissue deposition of the complement component C3b, and high serum levels of C5a [21]. Additionally, the sera of untreated GD patients exhibit very high C5a concentrations compared to those of healthy subjects [21]. Macrophages have receptors that interact with the potent opsonin C3b, and receptors that bind C5a to initiate inflammation. The complement cascade acts through two major pathways, classical (stimulated by antibodies) and alternative (stimulated by C3 hydrolysis). Complement component C5 initiates the “late” events of complement activation and is cleaved by C5 convertases into two fragments, C5a and C5b. C5a is an inflammatory peptide that is essential in the innate immune response, and also recruits adaptive immune cells through the induction of macrophage-secreted chemokines. C5b initiates the assembly of the MAC complex, a ring of complement proteins that form a lethal pore in the membrane of pathogens and other target cells.

It has been recently reported that C5a receptor-deficient mice were protected from GluCer accumulation in pulmonary macrophages, and that mouse survival was markedly increased, suggesting that C5a may play a role in GD pathogenesis [21]. The increased C5a levels detected in GD patient sera are thought to result from the presence of GluCer-specific auto-antibody immune complexes (IC) that activate the classical pathway of the complement system [21]. Elevated complement component C5a causes the release of TNF-α and other pro-inflammatory mediators by macrophages, leading to chronic inflammation [21,28,29,30,31]. Many studies have shown that the inflammatory process initiated by elevated TNF-α and the concomitant increase in ROS production can induce DNA damage, which may lead to cancer [28,29,32,33,34,35,36]. Thus, C5a-mediated inflammation in GD could, in the long-term, contribute to the development of cancers [20,21,22,37,38,39].

In addition to the adaptive immune response mediated by interactions of B cells and T cells with macrophages, there is an aberrant innate immune component caused by increased C5a levels alone, which is the focus of this study. We hypothesized that when GD iPSC-derived macrophages are exposed to recombinant human C5a (rC5a), these cells would recapitulate the exacerbated inflammatory response of Gaucher macrophages in patients. To study the consequences of complement-induced inflammation, we carried out transcriptomic analysis of rC5a-treated GD iPSC-derived macrophages. Our results revealed that in these cells, the pro-inflammatory action of the complement system is predominantly driven by the C5a-C5aR1 axis. Incubation of GD macrophages with exogenous rC5a led to elevated expression of TNF-α signaling pathway genes and dysregulation of the hepatic fibrosis pathway. Our results further suggest that C5a-C5aR1-inhibiting therapies may lessen the risk of potentiated inflammation, thereby diminishing the pathologies caused by overabundant C5a present in GD patient sera [21].

## 2. Results and Discussion

In this study. we used human iPSC-derived macrophages to investigate the role of C5a in innate inflammatory responses of GD vs. control macrophages (Supplemental Table S1). We previously showed that GD iPSC-derived macrophages are hypersensitive to LPS, and that they produce increased levels of IL-1β and TNF-α, recapitulating the elevation of these cytokines in sera of GD patients [16]. Here, we show that recombinant C5a activates a significant TNF-α inflammation response in human GD macrophages, which is similar to that seen in animal models of GD [21]. Specifically, TNF-α induction by C5a was previously demonstrated in dendritic cells co-cultured with CD4^+^ T cells from Gba1^9V/-^ knockout mice [21]. Below, we describe the dysregulation of downstream gene expression caused by activation of the C5a-C5aR1 axis, and show that these effects are directly caused by GCase deficiency. We also identify a set of differentially expressed genes (DEGs) between unstimulated GD and control macrophages. As global transcriptome changes have not been previously studied in human Gaucher macrophages or in the context of rC5a stimulation, our results represent an important step in understanding how C5a-C5aR1 axis stimulation contributes to chronic inflammation in GD patients.

As shown in Supplemental Figure S1, flow cytometry analysis showed that up to 95% of iPSC-derived macrophage were positive for the monocyte/macrophage lineage markers CD14 and CD68. All resting macrophages that were produced were phagocytic, and GD macrophages displayed normal C3b-mediated phagocytosis via the classical pathway (Supplemental Figure S2).

Since complement components C3b and C5a levels are both increased in GD mouse models, we sought to determine if their respective effector functions would be altered in human macrophages. We first compared Fcγ receptor- and C3b receptor-mediated phagocytosis. As shown in Supplemental Figure S2, phagocytic activity between control and GD macrophages was comparable in Fcγ- and complement-dependent assays. Therefore, if sufficient C3b is provided, then GD macrophages phagocytose normally. Given that complement component C3b-mediated phagocytosis was not defective, we next sought to determine if GD macrophages had an aberrant response to stimulation of the C5a-C5aR1 axis.

### 2.1. Recombinant C5a Induces an Elevated TNF-α-Mediated Inflammatory Response in GD Macrophages

**Figure 1:**
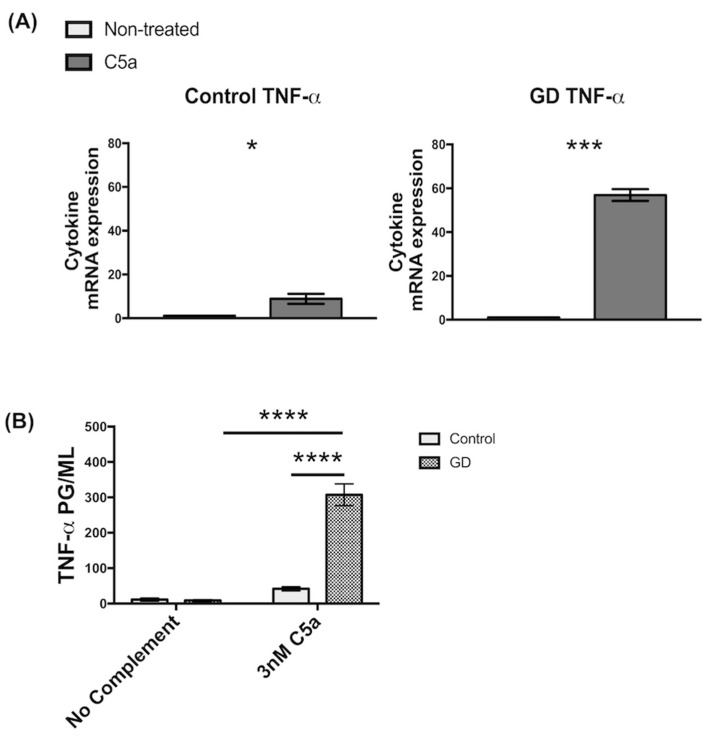
**GD macrophages have increased TNF-α gene expression and secretion in the presence of rC5a (A)** GD and control Inflammatory Gene Expression in iPSC-derived macrophages with 2 h of rC5a (30 nM) incubation. rC5a-stimulated cells were standardized to their respective unstimulated counterparts. Significance was determined using an independent-samples *t*-test. The primer sets that were used for the qRT-qPCR analysis (*n* = 3) are described in Supplemental Table S2, (**B**) ELISA of TNF-α in iPSC-derived macrophages with 8 h of rC5a (3 nM) incubation. Significance was determined using an ANOVA (*n* = 3), * *p* < 0.05, *** *p* < 0.001, **** *p* < 0.0001.

Based on the current literature, the only complement component that is markedly elevated in GD patient sera is C5a [21]. To determine if C5a plays a role in altered responses of GD macrophages, we examined if C5a had an effect on inflammation, its macrophage effector function. To this end, we incubated control and GD macrophages in media containing recombinant human C5a (rC5a) and analyzed their inflammatory responses. Quantitative RT-PCR analysis showed that rC5a incubation for 2 h caused a significant upregulation of inflammatory gene expression in both control and GD macrophages, but the increase in levels of Tumor Necrosis Factor alpha (TNF-α) mRNA were significantly higher in GD cells. The rC5a-mediated induction of TNF-α mRNA in control macrophages was ~9-fold, whereas in GD macrophages it was ~56-fold, representing a ~6-fold increase in sensitivity to rC5a (Figure 1A). ELISA assays were performed on culture supernatants to measure TNF-α cytokine secretion. After stimulation with rC5a for 8 h, GD macrophages were found to secrete higher levels of TNF-α into the culture medium than C5a-treated control macrophages (Figure 1B). The inflammatory response observed in GD macrophages was presumably initiated by C5a alone, independently of other upstream components in the classical pathway, since no additional complement proteins were exogenously introduced. From these experiments, we conclude that GD macrophages express and release significantly more TNF-α in response to stimulation by rC5a than controls.

**Figure 2:**
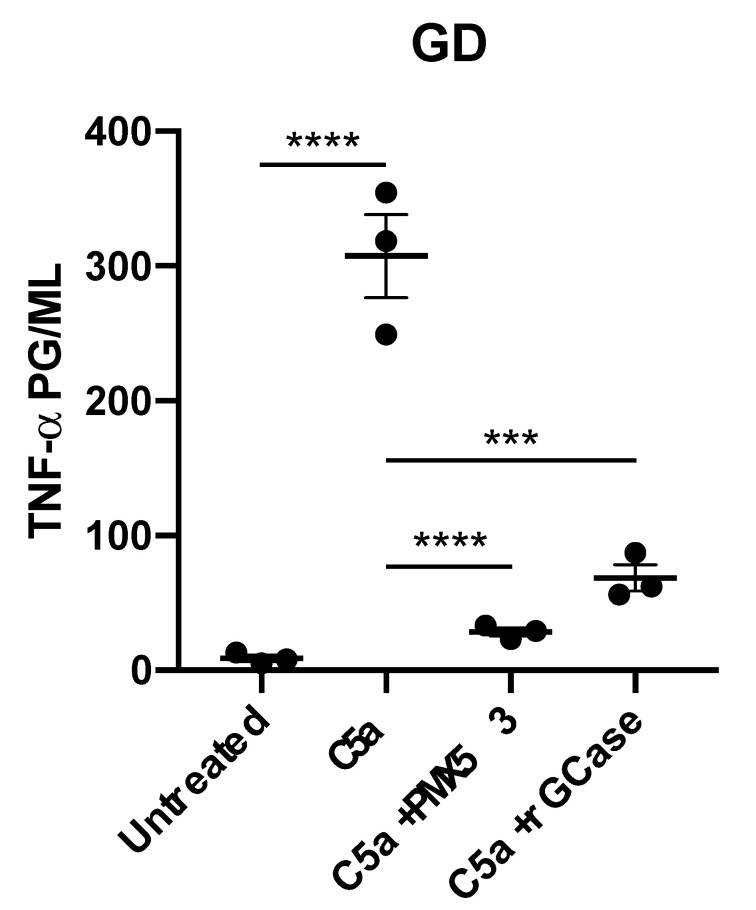
**C5aR1 antagonist or rGCase effectively prevent excess secretion of TNF-α.** GD macrophages were stimulated with rC5a (3 nM) in the presence or absence of PMX53 (50 nM) at 37 °C for 8 h. Recombinant GCase (0.24 U/mL) was added to the media every 24 h for 3 days prior to rC5a stimulation. The supernatant was collected and quantified for the expression of TNF-α by ELISA. Results are represented as the mean (*n* = 3; *** *p* < 0.001, **** *p* < 0.0001).

To determine if the increased expression of TNF-α by GD macrophages was triggered by the binding of rC5a to its receptor, C5aR1 was blocked by pretreatment of cells with PMX53, a C5aR1 antagonist, prior to stimulation with rC5a. As shown in Figure 2, C5aR1 blockade prevented the rC5a-induced increase in TNF-α cytokine secretion by GD macrophages. Incubation of the GD macrophages with recombinant GCase (rGCase) also prevented the rC5a-induced increase in TNF-α production, demonstrating that the rC5a effects observed were not an artifact of the iPSC system, but were indeed due to the loss of GCase in the mutant cells (Figure 2), which leads to substrate accumulation. Our results suggest that in the absence of functional GCase, rC5a stimulation triggers an abnormal increase in cytokine production, in agreement with the idea that GCase enzyme activity is required for a balanced inflammatory response of macrophages [18,40,41,42,43].

### 2.2. Microarray Analysis of GD Macrophages

**Figure 3:**
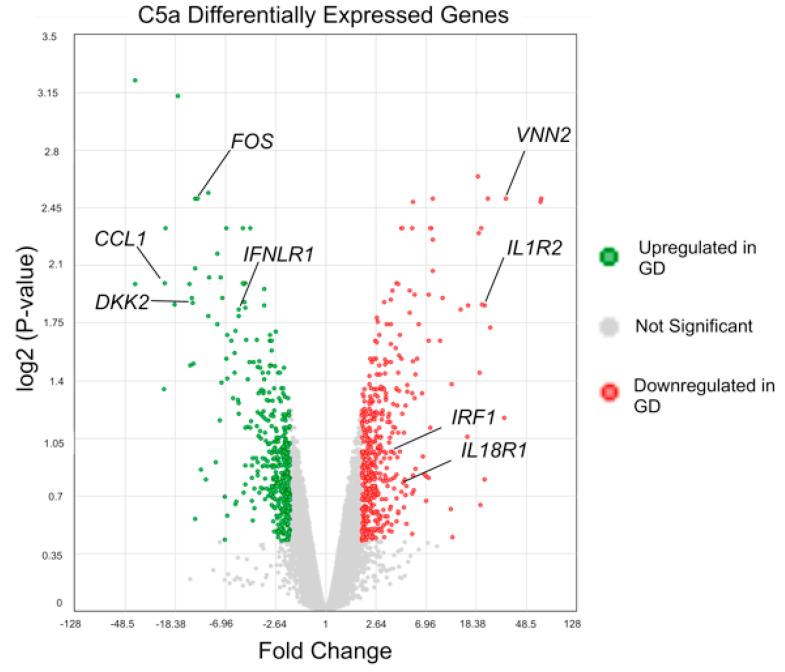
**Volcano plot of differentially expressed genes.** Volcano plot of differentially expressed genes (DEGs) in control vs. GD macrophages incubated with rC5a (*n* = 2).

**Figure 4:**
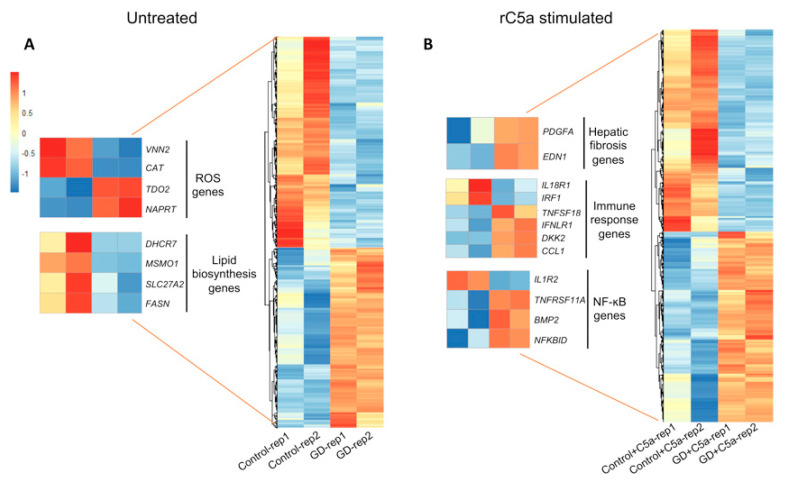
**Heat map of differentially expressed genes.** Heat map of Affymetrix microarray data showing differentially expressed genes in control vs. GD macrophages incubated without and with rC5a. (**A**)The heat map shows 940 genes, which were found differentially expressed between untreated control and GD when testing for a fold-change level of gene expression set at >2. (**B**) The heat map shows 855 genes differentially expressed during C5a-induced inflammation. In the heat map graphics, rows show individual genes. Duplicate samples are depicted in columns. Gene expression levels are displayed for each independent sample. Enlargements represent select genes of interest derived from the global “Untreated” and “rC5a-stimulated” heatmaps. Overexpression is shown in red, under-expression in blue (*n* = 2).

**Figure 5:**
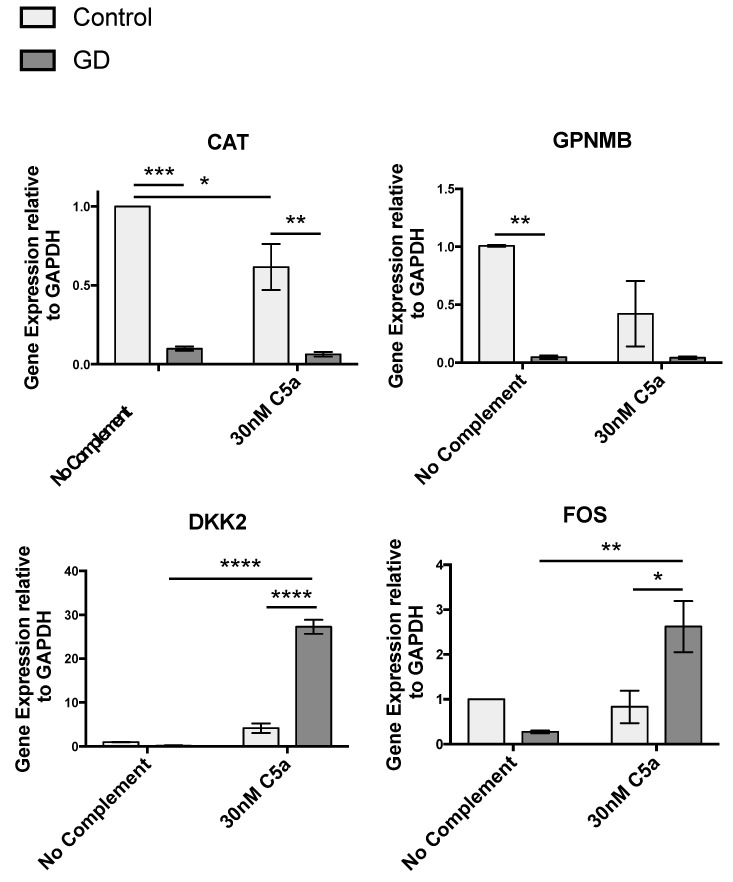
**qRT-PCR validation of select genes from the gene array.** qRT-PCR validation of genes selected from the microarray profile. Total RNA from the macrophage preparations was analyzed by qRT-PCR for the expression of a subset of genes randomly selected from those up- or down- regulated genes after 4 h of incubation with rC5a (30 nM). Expression changes were standardized to GAPDH. Results are presented as relative gene expression. (*n* = 3; * *p* < 0.05, ** *p* < 0.01, *** *p* < 0.001, **** *p* < 0.0001).

To further investigate the effects of increased sensitivity of GD macrophages to rC5a, we examined transcriptomic differences between control and GD macrophages before and after rC5a treatment. Our analyses indicate that after a 4 h incubation with rC5a, of the >24,000 genes analyzed, 855 genes were differentially expressed between control and GD macrophages (Figure 3 and Figure 4 and Supplemental Table S3A–C). The distribution of the Differentially Expressed Genes (DEGs) in the presence of rC5a is shown in the Volcano Plot in Figure 3, pinpointing the genes with the largest magnitude of fold-change that are considered statistically significant. The heat map shown in Figure 4 represents the total number of 440 downregulated DEGs and 415 upregulated DEGs found in the C5a-treated gene set. Gene Ontology analyses further compared untreated GD vs. control and GD-C5a vs. control-C5a and identified the top pathways with the highest number of DEGs in each group. The upper heatmap shows that some of the aforementioned DEGs in untreated GD vs. untreated controls (*VNN2*, *CAT*, *NAPRT*, and *TDO2*) are specifically involved in the production of nitric oxide and reactive oxygen species (Figure 4 and Supplemental Table S3A). Genes from lipid biosynthesis pathways from this group comparison (*DHCR7*, *MSMO1*, *SLC27A2*, and *FASN*) are also shown in Figure 4. The bottom heatmap shows that the top DEGs between GD-C5a vs. control-C5a are involved in hepatic fibrosis (*EDN1* and *PDGFA*), NF-κB (*IL1R2*, *TNFRSF11A*, *NFKBID*, and *BMP2*), and in immune responses (*IL18R1*, *IRF1*, *TNFSF18*, *IFNLR1*, *DKK2* and *CCL1*) (Figure 4). The results of this microarray analysis were validated by quantitative real-time PCR (qRT-PCR) of select genes, including *CAT*, *DKK2*, *FOS*, and *GPNMB* (Figure 5).

### 2.3. Differentially Expressed Genes between Untreated GD vs. Control Macrophages

#### 2.3.1. Redox Imbalance

Supplemental Table S3A shows a list of differentially expressed genes (DEGs) in unstimulated GD vs. control macrophages. These DEGs were principally associated with the oxidative-stress responses. In particular, *CAT* and *VNN2* were found to be more highly expressed in control macrophages and were among the genes with the highest relative fold-change. The *CAT* gene encodes catalase, a key antioxidant enzyme that protects aerobic cells from reactive oxygen species (ROS). GD patients are primed for oxidative response complications because they are known to have a severe redox imbalance partially caused by alterations in catalase activity [44]. While plasma from GD patients has elevated catalase enzymatic activity, GD RBCs have significantly lower catalase activity [18,45]. We found that unstimulated GD macrophages express significantly lower levels of *CAT*, the gene that encodes for catalase, than unstimulated control macrophages. Our data suggest that the low basal *CAT* mRNA expression in GD macrophages may contribute to reduced ROS detoxification (Supplemental Table S3C). Additionally, *VNN2* encodes an enzyme of the vascular non-inflammatory molecule (Vanin) family, reported to be involved in the oxidative stress response [46]. *NAPRT*, which is more highly expressed by GD macrophages, encodes nicotinate phosphoribosyl transferase, the first enzyme involved in the nicotinic acid (NA) pathway of cellular redox reactions [46]. Macrophages activated by oxidative stress can exhibit an increased or chronic inflammatory response [47]. As GD patients have oxidative response complications, our results appear to reflect the known redox imbalance in GD patients.

#### 2.3.2. Gene Array Analysis Indicates Dysregulated Cholesterol Metabolism in GD

Our analysis showed that several genes involved in cholesterol biosynthesis, fatty acid degradation, and lipoprotein metabolism were downregulated in GD macrophages (e.g., *DHCR7*, *MSMO1*, *SLC27A2*, *CYP51A1*, *FASN, APOC1*, and *APOE*) (Figure 4) [43]. Genetic defects in cholesterol metabolism are primarily associated with neuropathology. In particular, *DHCR7* and *MSMO1* were previously found to be down-regulated upon injection of mice with conduritol B-epoxide (CBE), an irreversible inhibitor of GCase that recapitulates a number of alterations found in GD [43]. Thus, the defective lipid biosynthesis and lipoprotein metabolism of human iPSC-macrophages also reflects known GD pathology.

#### 2.3.3. Gene Array Analysis Suggests Potential New Biomarkers for GD

In agreement with a recent publication by Lugowska et al. using GD fibroblasts [18], we found that GD macrophages exhibited significant up-regulation of a number of other immune response genes, including *IFIT1*, *IFIT2*, *MX1*, *OAS2*, and *LY6E* [18]. Future experiments will explore whether any of these genes could be used as potential new GD biomarkers.

### 2.4. rC5a-Stimulated GD vs. rC5a-Stimulated Control Macrophages

#### 2.4.1. Pathway Analysis

We then performed Gene Ontology analysis on GD and control macrophages incubated with and without exogenous rC5a. These Ingenuity Pathway analyses revealed that the genes most differentially expressed between rC5a-stimulated GD and rC5a-stimulated control macrophages were associated with hepatic fibrosis and immune signaling pathways (Supplemental Table S4).

#### 2.4.2. C5a Treatment of GD Macrophages Upregulates Genes Involved in Hepatic Fibrosis

The biological pathway with the highest number of log fold-change DEGs (Supplemental Tables S3 and S4) upon C5a treatment is involved in hepatic fibrosis, a well-studied, long-term clinical manifestation of GD [2,26,48,49]. Hepatomegaly and liver fibrosis are a common condition in GD patients, which is associated with infiltration of the liver by pathological macrophages [48]. Although ERT and SRT treatment improves hepatocellular damage, GD patients still exhibit mildly increased markers of hepatic damage and an increased rate of steatosis that are not completely corrected by these treatments [26].

Our analysis revealed that C5a strongly induced hepatic fibrosis pathway gene expression in both GD and control macrophages (*BCL2*, *CCL2*, *CCL3*, *CCND1*, *IKBKE*, *IL1B*, *IL1R2*, *IL1RN*, *IRAK2*, *ITGB3*, *MAP2K3*, *MYC*, and *RND3*). Of note, *EDN1* and *PDGFA*, which are specific drivers of hepatic fibrosis [50,51,52], were upregulated in rC5a-treated GD macrophages, but not rC5a-treated control cells. As macrophages are the most abundant immune cells in the liver, and they play a key role in injury and repair during liver disease [53], our results suggest that the increased susceptibility of GD macrophages to C5a dysregulation of genes involved in hepatic fibrosis may contribute to aggravation of this condition in GD patients [51,53,54].

#### 2.4.3. Inflammatory Gene Expression Profile Induced by rC5a in GD Macrophages

The proinflammatory cytokine TNF-α is critical in determining the outcome of a macrophage-mediated immune response [55]. We found that the highest relative fold-change in genes differentially expressed between rC5a-stimulated control and rC5a-stimulated GD macrophages corresponded to TNF-α and many other important genes involved in the immune response (Supplemental Tables S3B and S4). *TNF*, additional inflammatory cytokines, and cytokine regulators such as *CCL1*, *TNFRSF11A*, *IFNLR1*, *CCL24*, *TNFRSF10A*, *AREG*, and *IFITM3*, were all elevated in rC5a-induced GD macrophages. In particular, *CCL1* and *CCL24* primarily function in eosinophil and regulatory T cell trafficking. TNF receptors (*TNFRSF11A* and *TNFRSF10A*) are important regulators of leukocyte activation and promote the activation of Nuclear Factor kappa B (NF-κB) (Supplemental Table S3B). *IFNLR1* encodes an IFN-λ cytokine receptor complex that is important for antiviral immune defense and has been found to promote immune dysregulation [56]. *AREG*, a gene induced by TNF-α, encodes Amphiregulin, a growth factor that is associated with a number of inflammatory and autoimmune conditions [57,58]. Finally, *IFITM3*, another gene induced by TNF-α, encodes an antiviral protein that is critical for phagocytic endosome formation and disrupts intracellular cholesterol homeostasis [59,60]. TNF-α factors regulate two major downstream signaling pathways, NF-κB and mitogen-activated protein kinases (MAPK), implicating them as potential drivers of GD pathology and neuroinflammation [61,62,63,64,65]. Thus, rC5a activates a distinct set of inflammatory genes, preceded by high levels of TNF-α, suggesting that C5a contributes to chronic inflammation in GD through TNF-α pathway deregulation. We should note, however, that in future experiments additional patient lines will need to be analyzed in order to validate these conclusions.

#### 2.4.4. Wnt Signaling

The response to TNF also elicits cross-regulation with other important signaling pathways [66]. In the case of GD, NF-κB and Wnt signal dysregulation are of particular consequence for axonal guidance, lysosomal loss, and bone differentiation [67,68,69]. Our pathway analysis of C5a stimulation identified Wnt pathway genes, which are involved in development, arthritis, and cancer (Supplemental Table S4). Unstimulated GD macrophages have lower *WLS*, *LRP6*, and *CD44* expression compared to the unstimulated control (Supplemental Table S3A). *WLS* encodes for a transporter of Wnt to the plasma membrane and *LRP6* encodes for the major transmembrane Wnt co-receptor. Their reduced steady-state expression in unstimulated GD macrophages indicate a consistent downregulation of Wnt signaling activity compared to controls. We found that upon C5a treatment, GD macrophages increase expression of regulatory Wnt pathway genes, including *DKK2*, which can activate or inhibit canonical Wnt signaling, potentially leading to C5a-induced dysregulation of this pathway (Figure 3 and Figure 5, Supplemental Table S3B). Future studies will be needed to clarify the significance of the effects of C5a on Wnt signaling.

In summary, our results show that rC5a contributes to a unique innate immune response in human GD macrophages and identifies a number of potential new regulators that may be involved in aberrant immune responses. We also identified a set of differentially expressed genes in untreated GD macrophages that could be used as markers for GD. Our results provide further support for exploring the use of anti-C5a or anti-C5aR therapeutics as an adjunctive treatment option for GD. In addition to the C5 inhibitors that are currently in the clinic, a more specific therapy that targets the C5a protein fragment and does not inhibit terminal MAC complex formation may lessen the risk of infection. In this regard, C5a/C5aR inhibitors (Avacopan or Vilobelimab) may help prevent chronic inflammation in GD and ameliorate its long-term complications [27].

## 3. Materials and Methods

### 3.1. iPSC Lines and Directed Differentiation to Macrophages

In this study, we used iPSC lines derived from a patient with type 2 GD and from two healthy controls (Supplemental Table S1). These control and GD iPSC lines were used to generate monocyte-producing bioreactors as we previously described [70], with the modification that during differentiation of iPSCs into monocytes, 10 μM of Rock inhibitor was added to the embryoid body (EB) culture medium for 4 days.

Monocytes harvested from EBs were resuspended in macrophage differentiation medium (RPMI (Gibco, Waltham, MA, USA))/10% FBS (Hyclone, Logan, UT, USA), supplemented with 100 ng/mL hM-CSF (Miltenyi, Bergisch Gladbach, Germany), Glutamine (Gibco, Waltham, MA, USA), and Pen/Strep (Gibco, Waltham, MA, USA) and plated in chamber slides or 6-well plates for at least 5 days. All macrophages were analyzed within 10 days of plating.

The work with the human iPSC lines used in this study is considered non-human research; these iPSC lines are exempt under 45 CFR Part 46. Work with these iPSC lines was approved by the University of Maryland School of Medicine Institutional Review Board (IRB) on 15 July 2009 (HP-42545).

### 3.2. Flow Cytometry

Microarray analysis was carried out at the University of Maryland Greenebaum Comprehensive Cancer Center Flow Cytometry Shared Service (FCSS). Induced macrophages were fixed in paraformaldehyde, washed, and incubated in blocking buffer consisting of PBS, human Fc Block (0.5 mg/mL, BD), 8% FBS, and 0.01% sodium azide. Cells were then incubated with APC Mouse Anti-Human CD14, APC Mouse IgG2α, к Isotype Control, PE Mouse Anti-Human CD68, or PE Mouse IgG2b к Isotype control (BD, San Jose, CA, USA) in buffer containing PBS, 0.2% saponin, 8% FBS, and sodium azide, washed, and kept at 4 °C until fluorescence-activated cell sorting analysis. Data were acquired by flow cytometry using a BD FACS Canto II Cell Flow Cytometry System and analyzed using FCS Express software (De Novo, Pasadena, CA, USA).

### 3.3. Measurement of Cytokine Expression by qRT-PCR

Macrophages were incubated for 2 or 4 h with 30 nM rC5a as indicated. After incubation, macrophages were washed with PBS and total RNA was isolated using a RNeasy kit with on-column DNase I digestion (Qiagen, Germantown, MD, USA). RNA samples were quantified with a ND-1000 spectrophotometer and 65–80 ng of total RNA from macrophages was reverse-transcribed using the iScript cDNA synthesis kit (Biorad, Hercules, CA, USA). For quantitative RT-PCR analyses, primers for human *GAPDH*, *TNF-Alpha*, *DKK2*, *FOS*, *GPNMB*, and *CAT* were used (Supplemental Table S2). An Applied Biosystems 7500 Real-Time PCR system was used with Power SYBR Green Master mix (Applied Biosystems, Waltham, MA, USA) at an amplification profile of 95 °C for 10 min, followed by 40 cycles of 95 °C for 15 s, and 60 °C for 60 s. The specificity of all reactions was determined by melting curve analyses at the end of PCR cycles. Gene expression was calculated using the minus delta–delta Ct method (2^−ΔΔCt^) method by normalizing the threshold cycle (Ct) values against the reference gene GAPDH [67].

### 3.4. Measurement of Cytokine Secretion by ELISA

ELISAs were carried out at the Cytokine Core Laboratory at the Center for Innovative Biomedical Resources (CIBR) Genomics Core Facility at the University of Maryland. To measure TNF-α cytokine production, we used enzyme-linked immunosorbent analysis (ELISA). Monocytic cells were cultured in 12-well plates at a density of 2 × 10^5^ cells/well in 1.2 mL of macrophage differentiation medium per well for 5–10 days. On the day of analysis, cells were washed in PBS, and macrophage medium was replaced with medium containing 3 nM recombinant human C5a (rC5a) (Sino Biological, Beijing, China #10604-HNAE). After 8 h, macrophage culture supernatants were collected and assayed for the indicated cytokines by ELISA as described below. After an 8 h rC5a (3 nM) incubation, supernatant was collected and submitted to the University of Maryland Cytokine Core Laboratory for analysis. Where indicated, the C5aR antagonist, PMX53 (50 nM), (Sigma) was added concurrently with rC5a during the 8 h incubation period. Where indicated, GD iPSC macrophages were incubated with recombinant human glucocerebrosidase (rGCase) (Cerezyme^®^, Genzyme, Cambridge, MA, USA) at a concentration of 0.24 U/mL for 3 days prior to rC5a incubation. Cerezyme was obtained from patient infusion remnants. The cytokines were measured by two-antibody ELISA using biotin-strepavidin-peroxidase detection. Polystyrene plates Maxisorp (Nunc, Roskilde, Denmark) were coated with capture antibody in PBS overnight at 25 °C. The plates were washed 4 times with 50 mM Tris, 0.2% Tween-20, pH 7.0–7.5 and then blocked for 90 min at 25 °C with assay buffer (PBS containing 4% BSA (Sigma, St. Louis, MO, USA). Then, 50 µL of sample or standard prepared in assay buffer was added to each well and incubated at 37 °C for 2 h. The plates were washed 4 times and 100 µL of biotinylated detecting antibody in assay buffer was added and incubated for 1 h at 25 °C. After washing the plate 4 times, strepavidin-peroxidase polymer in casein buffer (Research Diagnostics Inc., Flanders, NJ, USA) was added and incubated at 25 °C for 30 min. The plate was washed 4 times and 100 µL of commercially prepared substrate (TMB; Dako, Carpinteria, CA, USA) was added and incubated at 25 °C for approximately 10–30 min. The reaction was stopped with 100 µL 2N HCl and the A450 (minus A650) was read on a microplate reader (Molecular Devices, San Jose, CA, USA). A curve was fitted to the cytokine standards using SoftMaxPro 7; Molecular Devices, San Jose, CA, USA) and cytokine concentration in each sample was calculated from the standard curve equation.

### 3.5. Microarray Analysis

Microarray analysis was carried out at the Center for Innovative Biomedical Resources (CIBR) Genomics Core Facility at the University of Maryland. Total RNA was extracted from macrophages using a RNeasy Mini kit (Qiagen, Hilden, Germany) according to the manufacturer’s protocol. After processing with DNase digestion (RNase-Free DNase set, Qiagen, Hilden, Germany) and clean-up procedures, RNA samples were quantified, aliquoted and stored at −80 °C until use. For quality control, RNA purity and integrity were evaluated by OD 260/280 ratio and analyzed on an Agilent 2100 Bioanalyzer (Agilent Technologies, Santa Clara, CA, USA). Five hundred ng of total RNA was reverse transcribed to cDNA using the GeneChip^TM^ WT PLUS Reagent Kit (ThermoFisher, Waltham, MA, USA). The quality of hybridization was monitored, and raw data were extracted using the software provided by the manufacturer (Illumina GenomeStudio v2011.1 (Gene Expression Module v1.9.0) (San Diego, CA, USA)). The overall chip performance was monitored using the Affymetrix Expression Console Software. The raw microarray profiling data was preprocessed and quartile-normalized using the Transcriptome Analysis Console Software (Version 4.0.1) (https://www.thermofisher.com/us/en/home/life-science/microarray-analysis/microarray-analysis-instruments-software-services/microarray-analysis-software/affymetrix-transcriptome-analysis-console-software.html, accessed on 1 June 2021). Ingenuity pathway analysis was used to identify enriched Gene Ontology processes. To identify differentially expressed mRNAs between groups, an ANOVA with an eBayes test was used. Genes with fold-change greater than 2 and a *p*-value < 0.05 were considered significant.

### 3.6. Phagocytosis Assays

#### 3.6.1. Fcγ-Mediated Phagocytosis

Sheep red blood cells (SRBCs) (Lampire Biologicals, Pipersville, PA, USA) were washed several times with saline and opsonized by incubation with rabbit anti-SRBC IgG (2 μL per 1 × 10^9^ cells in 1 mL) (MP Biomedicals, Santa Ana, CA, USA) at 37 °C for 1 h with gentle agitation. RBCs were washed with saline to remove the unbound antibody. Macrophages cultured in 24-well plates at 1 × 10^5^ cells per well for 5 d were incubated with opsonized RBC at a ratio of 25:1 at 37 °C for 1.5 h. After incubation, the cultures were washed to remove excess RBC and ammonium-chloride-potassium lysing buffer (Lonza, Walkersville, MD, USA) was added to remove RBC attached to the surface of the macrophages. A minimum of 300 macrophages per field was scored for the presence of ingested RBC. Non-opsonized RBCs were used as a negative control.

#### 3.6.2. Complement Receptor-Mediated Phagocytosis

Five mL of SRBCs were washed several times with saline. SRBCs were then washed once with GVB + Calcium + Magnesium (GVB++) buffer (CompTech, Tyler, TX, USA) and resuspended in 5 mL of GVB++ solution. SRBCs were counted with a hemocytometer and the concentration was adjusted to 1 × 10^9^/mL. SRBCs were opsonized by incubation with rabbit anti-SRBC IgM (6 μL per 1 × 10^9^ cells in 1 mL) (MP Biomedicals, Santa Ana, CA) at 37 °C for 30 min with gentle agitation as previously described [71]. Then, SRBCs were washed with PBS to remove the unbound antibody. Next, SRBCs were resuspended in 1 mL of GVB++ buffer with 50 μL of C5-depleted serum (CompTech, Tyler, TX, USA at 37 °C for 30 min. Macrophages that were cultured in 24-well plates at 1 × 10^5^ cells per well for 5 d were incubated with the SRBCs opsonized with IgM and complement to measure binding mediated by complement receptors at a ratio of 25:1 at 37 °C for 1.5 h. After incubation, the cultures were washed as described above to remove excess SRBC, and ammonium-chloride-potassium lysing buffer (Lonza, Walkersville, MD, USA) was added to remove SRBC still attached on the surface of the macrophages. A minimum of 300 macrophages per field was scored for the presence of ingested SRBC. IgM-opsonized SRBCs incubated without complement were used as a negative control.

### 3.7. Statistics

For Figure 1A, we performed an unpaired *t*-test on the logarithmic transformed measurements. We used Levene’s test for equal variances in the two groups compared (controls vs. C5a treated cells). In the case of the TNFα controls, this led us to use the independent-samples *t*-test without assuming equal variances, yielding *p* = 0.012. For the GD cells, the equal variance assumption was not rejected, and in this case an equal-variance, independent-samples *t*-test yielded *p* < 0.001. Data analyses were expressed as the mean ± SEM and were carried out using an ANOVA with multiple comparisons where appropriate. *p* values less than 0.05 (*p* < 0.05) were considered statistically significant. Calculations were performed using GraphPad Prism 9 software.

## Data Availability

All the data supporting the reported results are provided in the main body of the Article or Supplementary Materials. All datasets generated in this this study are publicly available at the Gene Expression Omnibus (GEO), accession number GSE183484.

## Supplementary Materials

The following are available online at https://www.mdpi.com/article/10.3390/ijms22189912/s1.

## References

[1] National Human Genome Research Institute About Gaucher Disease. http://www.genome.gov/25521505.

[2] Stirnemann J., Belmatoug N., Camou F., Serratrice C., Froissart R., Caillaud C., Levade T., Astudillo L., Serratrice J., Brassier A. (2017). A Review of Gaucher Disease Pathophysiology, Clinical Presentation and Treatments. Int. J. Mol. Sci..

[3] Sheth J., Bhavsar R., Mistri M., Pancholi D., Bavdekar A., Dalal A., Ranganath P., Girisha K.M., Shukla A., Phadke S. (2019). Gaucher disease: Single gene molecular characterization of one-hundred Indian patients reveals novel variants and the most prevalent mutation. BMC Med. Genet..

[4] Stenson P.D. (2020). The Human Gene Mutation Database (HGMD). Hum. Genet..

[5] Mignot C., Gelot A., De Villemeur T.B. (2013). Gaucher disease. Handb. Clin. Neurol..

[6] Mucci J.M., Rozenfeld P. (2015). Pathogenesis of Bone Alterations in Gaucher Disease: The Role of Immune System. J. Immunol. Res..

[7] Burstein Y., Rechavi G., Rausen A.R., Frisch B., Spirer Z. (1985). Association of Gaucher’s disease and lymphoid malignancy in 2 children. Scand. J. Haematol..

[8] Hawkesford M.P., Bowey A.J., Rao J., Meara N.J. (2011). Synchronous presentation of Gaucher disease and solitary plasmacytoma with progression to multiple myeloma. Scott. Med. J..

[9] de Fost M., Vom Dahl S., Weverling G.J., Brill N., Brett S., Haussinger D., Hollak C.E. (2006). Increased incidence of cancer in adult Gaucher disease in Western Europe. Blood Cells Mol. Dis..

[10] Shoenfeld Y., Gallant L.A., Shaklai M., Livni E., Djaldetti M., Pinkhas J. (1982). Gaucher’s disease: A disease with chronic stimulation of the immune system. Arch. Pathol. Lab. Med..

[11] Rosenbloom B.E., Weinreb N.J., Zimran A., Kacena K.A., Charrow J., Ward E. (2005). Gaucher disease and cancer incidence: A study from the Gaucher Registry. Blood.

[12] Akiyama T., Sato S., Ko S.B.H., Sano O., Sato S., Saito M., Nagai H., Ko M.S.H., Iwata H. (2021). Synthetic mRNA-based differentiation method enables early detection of Parkinson’s phenotypes in neurons derived from Gaucher disease-induced pluripotent stem cells. Stem Cells Transl. Med..

[13] Srikanth M.P., Jones J.W., Kane M., Awad O., Park T.S., Zambidis E.T., Feldman R.A. (2021). Elevated glucosylsphingosine in Gaucher disease induced pluripotent stem cell neurons deregulates lysosomal compartment through mammalian target of rapamycin complex 1. Stem Cells Transl. Med..

[14] Rosenbloom B.E., Weinreb N.J. (2013). Gaucher disease: A comprehensive review. Crit. Rev. Oncog..

[15] National Library of Medicine: National Institutes of Health. Genetics Home Reference: Gaucher Disease. http://ghr.nlm.nih.gov/condition/gaucher-disease.

[16] Panicker L.M., Miller D., Awad O., Bose V., Lun Y., Park T.S., Zambidis E.T., Sgambato J.A., Feldman R.A. (2014). Gaucher iPSC-derived macrophages produce elevated levels of inflammatory mediators and serve as a new platform for therapeutic development. Stem Cells.

[17] Deganuto M., Pittis M.G., Pines A., Dominissini S., Kelley M.R., Garcia R., Quadrifoglio F., Bembi B., Tell G. (2007). Altered intracellular redox status in Gaucher disease fibroblasts and impairment of adaptive response against oxidative stress. J. Cell. Physiol..

[18] Lugowska A., Hetmanczyk-Sawicka K., Iwanicka-Nowicka R., Fogtman A., Ciesla J., Purzycka-Olewiecka J.K., Sitarska D., Ploski R., Filocamo M., Lualdi S. (2019). Gene expression profile in patients with Gaucher disease indicates activation of inflammatory processes. Sci. Rep..

[19] Van Rossum A., Holsopple M. (2016). Enzyme Replacement or Substrate Reduction? A Review of Gaucher Disease Treatment Options. Hosp. Pharm..

[20] Mistry P.K., Taddei T., vom Dahl S., Rosenbloom B.E. (2013). Gaucher disease and malignancy: A model for cancer pathogenesis in an inborn error of metabolism. Crit. Rev. Oncog..

[21] Pandey M.K., Burrow T.A., Rani R., Martin L.J., Witte D., Setchell K.D., McKay M.A., Magnusen A.F., Zhang W., Liou B. (2017). Complement drives glucosylceramide accumulation and tissue inflammation in Gaucher disease. Nature.

[22] Dubot P., Astudillo L., Therville N., Sabourdy F., Stirnemann J., Levade T., Andrieu-Abadie N. (2020). Are Glucosylceramide-Related Sphingolipids Involved in the Increased Risk for Cancer in Gaucher Disease Patients? Review and Hypotheses. Cancers.

[23] Limgala R.P., Goker-Alpan O. (2020). Effect of Substrate Reduction Therapy in Comparison to Enzyme Replacement Therapy on Immune Aspects and Bone Involvement in Gaucher Disease. Biomolecules.

[24] Regenboog M., van Dussen L., Verheij J., Weinreb N.J., Santosa D., Vom Dahl S., Haussinger D., Muller M.N., Canbay A., Rigoldi M. (2018). Hepatocellular carcinoma in Gaucher disease: An international case series. J. Inherit. Metab. Dis..

[25] Taddei T.H., Kacena K.A., Yang M., Yang R., Malhotra A., Boxer M., Aleck K.A., Rennert G., Pastores G.M., Mistry P.K. (2009). The underrecognized progressive nature of N370S Gaucher disease and assessment of cancer risk in 403 patients. Am. J. Hematol..

[26] Starosta R.T., Vairo F.P.E., Dornelles A.D., Basgalupp S.P., Siebert M., Pedroso M.L.A., Cerski C.T.S., Alvares-da-Silva M.R., Schwartz I.V.D. (2020). Liver involvement in patients with Gaucher disease types I and III. Mol. Genet. Metab. Rep..

[27] Horiuchi T., Tsukamoto H. (2016). Complement-targeted therapy: Development of C5- and C5a-targeted inhibition. Inflamm. Regen..

[28] Nair S., Sng J., Boddupalli C.S., Seckinger A., Chesi M., Fulciniti M., Zhang L., Rauniyar N., Lopez M., Neparidze N. (2018). Antigen-mediated regulation in monoclonal gammopathies and myeloma. JCI Insight.

[29] Nair S., Branagan A.R., Liu J., Boddupalli C.S., Mistry P.K., Dhodapkar M.V. (2016). Clonal Immunoglobulin against Lysolipids in the Origin of Myeloma. N. Engl. J. Med..

[30] Ilan Y. (2019). Beta-Glycosphingolipids as Mediators of Both Inflammation and Immune Tolerance: A Manifestation of Randomness in Biological Systems. Front. Immunol..

[31] van Eijk M., Ferraz M.J., Boot R.G., Aerts J. (2020). Lyso-glycosphingolipids: Presence and consequences. Essays Biochem..

[32] Perillo B., Di Donato M., Pezone A., Di Zazzo E., Giovannelli P., Galasso G., Castoria G., Migliaccio A. (2020). ROS in cancer therapy: The bright side of the moon. Exp. Mol. Med..

[33] Colotta F., Allavena P., Sica A., Garlanda C., Mantovani A. (2009). Cancer-related inflammation, the seventh hallmark of cancer: Links to genetic instability. Carcinogenesis.

[34] Cowan G., Weston-Bell N.J., Bryant D., Seckinger A., Hose D., Zojer N., Sahota S.S. (2015). Massive parallel IGHV gene sequencing reveals a germinal center pathway in origins of human multiple myeloma. Oncotarget.

[35] Cerutti P.A. (1994). Oxy-radicals and cancer. Lancet.

[36] Weitzman S.A., Gordon L.I. (1990). Inflammation and cancer: Role of phagocyte-generated oxidants in carcinogenesis. Blood.

[37] Yoneda M., Imamura R., Nitta H., Taniguchi K., Saito F., Kikuchi K., Ogi H., Tanaka T., Katabuchi H., Nakayama H. (2019). Enhancement of cancer invasion and growth via the C5a-C5a receptor system: Implications for cancer promotion by autoimmune diseases and association with cervical cancer invasion. Oncol. Lett..

[38] Medler T.R., Murugan D., Horton W., Kumar S., Cotechini T., Forsyth A.M., Leyshock P., Leitenberger J.J., Kulesz-Martin M., Margolin A.A. (2018). Complement C5a Fosters Squamous Carcinogenesis and Limits T Cell Response to Chemotherapy. Cancer Cell.

[39] Zhang R., Liu Q., Li T., Liao Q., Zhao Y. (2019). Role of the complement system in the tumor microenvironment. Cancer Cell Int..

[40] Mizukami H., Mi Y., Wada R., Kono M., Yamashita T., Liu Y., Werth N., Sandhoff R., Sandhoff K., Proia R.L. (2002). Systemic inflammation in glucocerebrosidase-deficient mice with minimal glucosylceramide storage. J. Clin. Investig..

[41] Stricklett P.K., Hughes A.K., Kohan D.E. (2005). Inhibition of p38 mitogen-activated protein kinase ameliorates cytokine up-regulated shigatoxin-1 toxicity in human brain microvascular endothelial cells. J. Infect. Dis..

[42] Karasu E., Demmelmaier J., Kellermann S., Holzmann K., Kohl J., Schmidt C.Q., Kalbitz M., Gebhard F., Huber-Lang M.S., Halbgebauer R. (2020). Complement C5a Induces Pro-inflammatory Microvesicle Shedding in Severely Injured Patients. Front. Immunol..

[43] Vardi A., Ben-Dor S., Cho S.M., Kalinke U., Spanier J., Futerman A.H. (2020). Mice defective in interferon signaling help distinguish between primary and secondary pathological pathways in a mouse model of neuronal forms of Gaucher disease. J. Neuroinflamm..

[44] Kartha R.V., Terluk M.R., Brown R., Travis A., Mishra U.R., Rudser K., Lau H., Jarnes J.R., Cloyd J.C., Weinreb N.J. (2020). Patients with Gaucher disease display systemic oxidative stress dependent on therapy status. Mol. Genet. Metab. Rep..

[45] de la Mata M., Cotan D., Oropesa-Avila M., Garrido-Maraver J., Cordero M.D., Villanueva Paz M., Delgado Pavon A., Alcocer-Gomez E., de Lavera I., Ybot-Gonzalez P. (2015). Pharmacological Chaperones and Coenzyme Q10 Treatment Improves Mutant beta-Glucocerebrosidase Activity and Mitochondrial Function in Neuronopathic Forms of Gaucher Disease. Sci. Rep..

[46] (2018). Database resources of the National Center for Biotechnology Information. Nucleic Acids Res..

[47] Kirkham P. (2007). Oxidative stress and macrophage function: A failure to resolve the inflammatory response. Biochem. Soc. Trans..

[48] Lachmann R.H., Wight D.G., Lomas D.J., Fisher N.C., Schofield J.P., Elias E., Cox T.M. (2000). Massive hepatic fibrosis in Gaucher’s disease: Clinico-pathological and radiological features. QJM.

[49] Bohte A.E., van Dussen L., Akkerman E.M., Nederveen A.J., Sinkus R., Jansen P.L., Stoker J., Hollak C.E. (2013). Liver fibrosis in type I Gaucher disease: Magnetic resonance imaging, transient elastography and parameters of iron storage. PLoS ONE.

[50] Rodriguez-Pascual F., Busnadiego O., Gonzalez-Santamaria J. (2014). The profibrotic role of endothelin-1: Is the door still open for the treatment of fibrotic diseases?. Life Sci..

[51] Thieringer F., Maass T., Czochra P., Klopcic B., Conrad I., Friebe D., Schirmacher P., Lohse A.W., Blessing M., Galle P.R. (2008). Spontaneous hepatic fibrosis in transgenic mice overexpressing PDGF-A. Gene.

[52] Wang Q., Chou X., Guan F., Fang Z., Lu S., Lei J., Li Y., Liu W. (2017). Enhanced Wnt Signalling in Hepatocytes is Associated with Schistosoma japonicum Infection and Contributes to Liver Fibrosis. Sci. Rep..

[53] Wen Y., Lambrecht J., Ju C., Tacke F. (2021). Hepatic macrophages in liver homeostasis and diseases-diversity, plasticity and therapeutic opportunities. Cell. Mol. Immunol..

[54] Lu J.W., Liao C.Y., Yang W.Y., Lin Y.M., Jin S.L., Wang H.D., Yuh C.H. (2014). Overexpression of endothelin 1 triggers hepatocarcinogenesis in zebrafish and promotes cell proliferation and migration through the AKT pathway. PLoS ONE.

[55] Parameswaran N., Patial S. (2010). Tumor necrosis factor-alpha signaling in macrophages. Crit. Rev. Eukaryot. Gene Expr..

[56] Goel R.R., Wang X., O’Neil L.J., Nakabo S., Hasneen K., Gupta S., Wigerblad G., Blanco L.P., Kopp J.B., Morasso M.I. (2020). Interferon lambda promotes immune dysregulation and tissue inflammation in TLR7-induced lupus. Proc. Natl. Acad. Sci. USA.

[57] Zaiss D.M.W., Gause W.C., Osborne L.C., Artis D. (2015). Emerging functions of amphiregulin in orchestrating immunity, inflammation, and tissue repair. Immunity.

[58] Platen C., Dreschers S., Wappler J., Ludwig A., Dusterhoft S., Reiss L.K., Orlikowsky T.W. (2019). Amphiregulin Regulates Phagocytosis-Induced Cell Death in Monocytes via EGFR and the Bcl-2 Protein Family. Mediat. Inflamm..

[59] Spence J.S., He R., Hoffmann H.H., Das T., Thinon E., Rice C.M., Peng T., Chandran K., Hang H.C. (2019). IFITM3 directly engages and shuttles incoming virus particles to lysosomes. Nat. Chem. Biol..

[60] Lee J., Robinson M.E., Ma N., Artadji D., Ahmed M.A., Xiao G., Sadras T., Deb G., Winchester J., Cosgun K.N. (2020). IFITM3 functions as a PIP3 scaffold to amplify PI3K signalling in B cells. Nature.

[61] Kitatani K., Wada M., Perry D., Usui T., Sun Y., Obeid L.M., Yaegashi N., Grabowski G.A., Hannun Y.A. (2015). Activation of p38 Mitogen-Activated Protein Kinase in Gaucher’s Disease. PLoS ONE.

[62] Aflaki E., Moaven N., Borger D.K., Lopez G., Westbroek W., Chae J.J., Marugan J., Patnaik S., Maniwang E., Gonzalez A.N. (2016). Lysosomal storage and impaired autophagy lead to inflammasome activation in Gaucher macrophages. Aging Cell.

[63] Zhu J., Jiang L., Liu Y., Qian W., Liu J., Zhou J., Gao R., Xiao H., Wang J. (2015). MAPK and NF-kappaB pathways are involved in bisphenol A-induced TNF-alpha and IL-6 production in BV2 microglial cells. Inflammation.

[64] Bernhardi R.V., Binder M.D., Hirokawa N., Windhorst U. (2009). Neurodegenerative Diseases–MAPK Signalling Pathways in Neuroinflammation. Encyclopedia of Neuroscience.

[65] Flood P.M., Qian L., Peterson L.J., Zhang F., Shi J.S., Gao H.M., Hong J.S. (2011). Transcriptional Factor NF-kappaB as a Target for Therapy in Parkinson’s Disease. Parkinson Dis..

[66] Du Q., Geller D.A. (2010). Cross-Regulation Between Wnt and NF-kappaB Signaling Pathways. Onco Ther..

[67] Dasgupta N., Xu Y.H., Li R., Peng Y., Pandey M.K., Tinch S.L., Liou B., Inskeep V., Zhang W., Setchell K.D. (2015). Neuronopathic Gaucher disease: Dysregulated mRNAs and miRNAs in brain pathogenesis and effects of pharmacologic chaperone treatment in a mouse model. Hum. Mol. Genet..

[68] Awad O., Panicker L.M., Deranieh R.M., Srikanth M.P., Brown R.A., Voit A., Peesay T., Park T.S., Zambidis E.T., Feldman R.A. (2017). Altered Differentiation Potential of Gaucher’s Disease iPSC Neuronal Progenitors due to Wnt/beta-Catenin Downregulation. Stem Cell Rep..

[69] Costa R., Bellesso S., Lualdi S., Manzoli R., Pistorio V., Filocamo M., Moro E. (2020). A transcriptional and post-transcriptional dysregulation of Dishevelled 1 and 2 underlies the Wnt signaling impairment in type I Gaucher disease experimental models. Hum. Mol. Genet..

[70] Panicker L.M., Miller D., Park T.S., Patel B., Azevedo J.L., Awad O., Masood M.A., Veenstra T.D., Goldin E., Stubblefield B.K. (2012). Induced pluripotent stem cell model recapitulates pathologic hallmarks of Gaucher disease. Proc. Natl. Acad. Sci. USA.

[71] Mosser D.M., Zhang X. (2011). Measuring opsonic phagocytosis via Fcgamma receptors and complement receptors on macrophages. Curr. Protoc. Immunol..

