# Supplementary material for: C5a Activates a Pro-Inflammatory Gene Expression Profile in Human Gaucher iPSC-Derived Macrophages
Source: Int J Mol Sci. 2021 Sep 14;22(18):9912. doi: 10.3390/ijms22189912 (PMC8466165; doi:10.3390/ijms22189912)
Supplement: Supplementary file 1 [file ijms-22-09912-s001.zip › ijms-1310008 Supplemental Figure and Table titles, and Figure and Table legends.pdf]

Supplemental Figure S1: Marker analysis of iPSC-derived macrophages. Flow Cytometry analysis of control and GD macrophages using antibodies against CD-14 and CD-68 after 7 days in differentiation media (See Materials and Methods), red: isotype controls, blue, reactive antibodies.

Supplemental Figure S2. There is no difference in Fc-gamma receptor-dependent or Complement receptor-mediated phagocytosis between the GD and control macrophages. Fc-gamma receptor-dependent phagocytosis (left) and complement receptor-mediated phagocytosis (right) were assayed as described in the Materials & Methods (*n*=3*;* ns=not significant*).*

Supplemental Table S1. Cell lines used in this study.

Supplemental Table S2. Primer sets used for qRT-PCR analysis

Supplemental Tables S3A, S3B, S3C, and S3D. Gene array analysis. Gene array analysis shows differential gene expression between (**A**) unstimulated GD *vs.* unstimulated control macrophages (**B**) GD-C5a *vs.* control-C5a macrophages incubated with rC5a (30 nM) for 4 h (**C**) unstimulated control *vs.* control-C5a (**D**) unstimulated GD *vs.* GD-C5a. Supplemental Tables 3A, 3B, 3C, and 3D are presented as Excel files in Supplementary information.

Supplemental Tables S4A, S4B, S4C, and S4D. Gene Ontology (GO) analysis. GO analysis of **(A)** unstimulated GD *vs.* unstimulated control macrophages, (**B**) GD-C5a *vs.* control-C5a macrophages, (**C**) unstimulated control *vs.* control-C5a, (**D**) unstimulated GD *vs.* GD-C5a. Supplemental Tables 4A, 4B, 4C, and 4D are presented as Excel files in Supplementary information.
