# Supplementary material for: C5a Activates a Pro-Inflammatory Gene Expression Profile in Human Gaucher iPSC-Derived Macrophages
Source: Int J Mol Sci. 2021 Sep 14;22(18):9912. doi: 10.3390/ijms22189912 (PMC8466165; doi:10.3390/ijms22189912)
Supplement: Supplementary file 1 [file ijms-22-09912-s001.zip › Supplemental Table S1.pdf]

Types	Name of hiPSCs used	Genotypes
Control a	DF4-7T.A	WT
Control b	MJiPSC	WT
Type 2 GD	MNG-10-257	L444P/Rec*NciI*
