# Supplementary material for: C5a Activates a Pro-Inflammatory Gene Expression Profile in Human Gaucher iPSC-Derived Macrophages
Source: Int J Mol Sci. 2021 Sep 14;22(18):9912. doi: 10.3390/ijms22189912 (PMC8466165; doi:10.3390/ijms22189912)
Supplement: Supplementary file 1 [file ijms-22-09912-s001.zip › Supplemental Table S2.pdf]

Transcript Name	Primers, forward and Reverse	Expected size (bp), ref
Human GAPDH_for	5’- GTCGCTGTTGAAGTCAGAGG -3’	289, [69]
Human GAPDH_rev	5’- GAAACTGTGGCGTGATGG -3’	
Human TNF_for	5’- CACTTTGGAGTGATCGGCC -3’	144, [14]
Human TNF_rev	5’- CTCAGCTTGAGGGTTTGCTACAAC -3’	
Human DKK2_for	5’- AGTACCCGCTGCAATAATGG -3’	243, [70]
Human DKK2_rev	5’- GAAATGACGAGCACAGCAAA -3’	
Human FOS_for	5’- CTGGCGTTGTGAAGACCAT -3’	109, [71]
Human FOS_rev	5’- TCCCTTCGGATTCTCCTTTT -3’	
Human GPNMB_for	5’- TCCAGATGACAGACGTCCTGATG -3’	135, [72]
Human GPNMB_rev	5’- TCTGGGTGATCTCGCAGGTG -3’	
Human CAT_for	5’- TAAGACTGACCAGGGCATC -3’	201, [73]
Human CAT_rev	5’- CAAACCTTGGTGAGATCGAA -3’	
