# Supplementary figures and images for: C5a Activates a Pro-Inflammatory Gene Expression Profile in Human Gaucher iPSC-Derived Macrophages
Source: Int J Mol Sci. 2021 Sep 14;22(18):9912. doi: 10.3390/ijms22189912 (PMC8466165; doi:10.3390/ijms22189912)

**Control Macrophages**

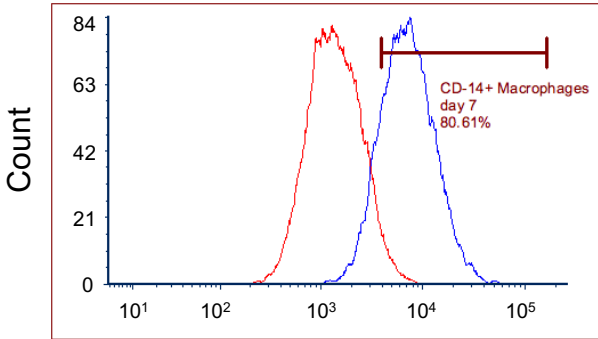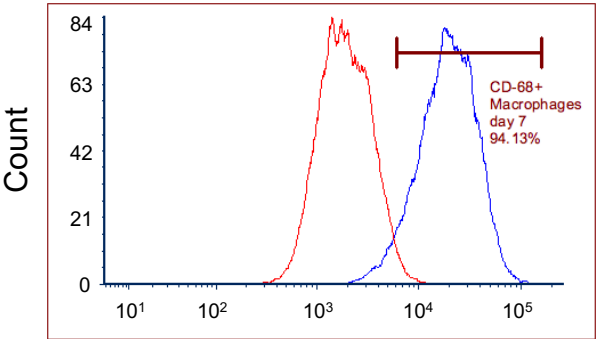

**GD Macrophages**

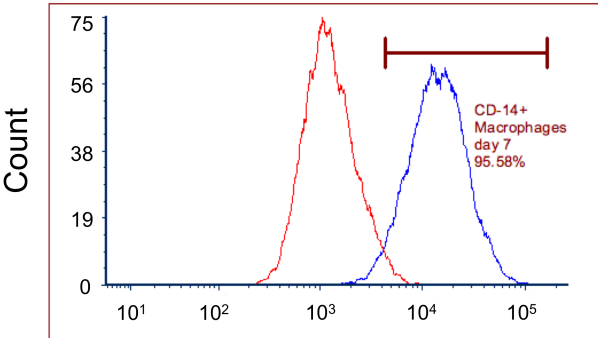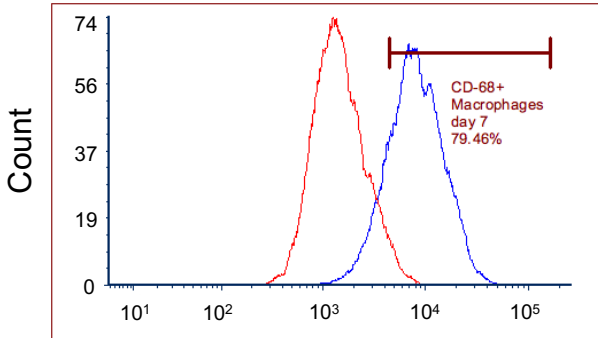

Supplement: Supplementary file 1 [file ijms-22-09912-s001.zip › Suppl Figure S1.pdf]

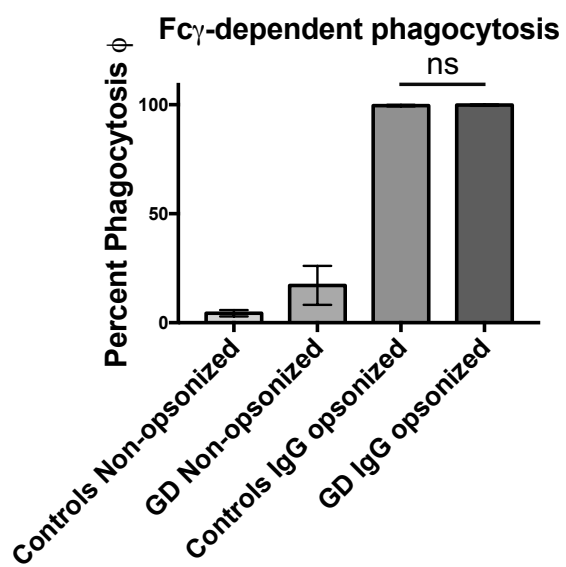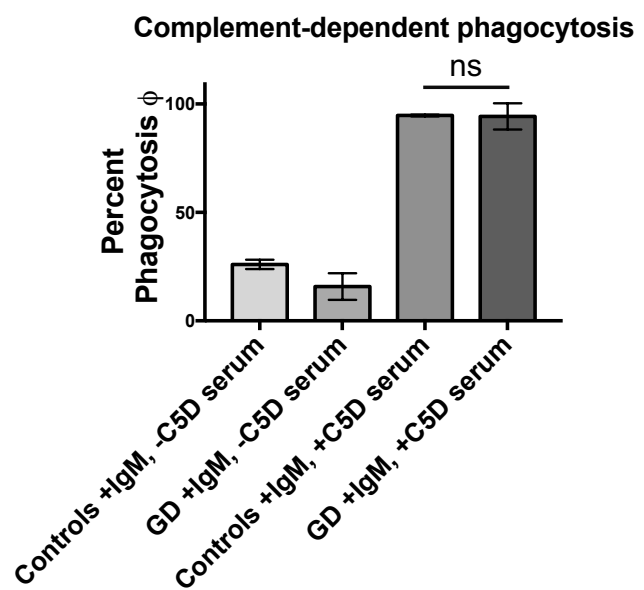

Supplement: Supplementary file 1 [file ijms-22-09912-s001.zip › Suppl Figure S2.pdf]
